# Individuals’ number of children is associated with benevolent sexism

**DOI:** 10.1371/journal.pone.0252194

**Published:** 2021-05-27

**Authors:** Chris K. Deak, Matthew D. Hammond, Chris G. Sibley, Joseph Bulbulia

**Affiliations:** 1 School of Psychology, Victoria University of Wellington, Wellington, New Zealand; 2 School of Psychology, University of Auckland, Auckland, New Zealand; National Taiwan University, TAIWAN

## Abstract

Is having children related to benevolent sexism? Two theoretical accounts—benevolent sexism as role justification and benevolent sexism as a mating strategy—suggest the possibility of a positive and bidirectional association. Gender disparities in childrearing could prompt inequality-justifying endorsement of benevolent sexism *and/or* endorsing benevolent sexism could promote traditional gender roles that facilitate having more children. We assessed the bidirectional associations between individuals’ number of children and their endorsement of benevolent sexism over a two-year period in a large national panel sample of New Zealanders (*N* = 6,017). Zero-inflated structural equation modeling indicated that having a greater number of children was associated with stronger endorsement of benevolent sexism two years later, but no evidence emerged for the reverse direction. This study illustrated ways to tentatively test predictions of theoretical accounts on sexism and identified new, though small, evidence for the role justification perspective.

## Introduction

Investment in childrearing creates new demands for parents which coincide with increased socioeconomic inequalities. For example, parenthood is associated with declines in women’s paid labor, earnings and career opportunities as well as increases in women’s unpaid labor and men’s paid labor and earnings [1–5]. According to ambivalent sexism theory, this inequality is linked with endorsement of *benevolent sexism*—subjectively positive and patronizing beliefs—toward women who invest in relationship-oriented and childrearing roles [6–10]. Two contrasting but complementary theoretical accounts of benevolent sexism suggest that there should be a positive and bidirectional link between how many children individuals have and their endorsement of benevolent sexism. A role justification perspective suggests that gender beliefs emerge from existing inequalities in social roles [11, 12], and if parenthood coincides with experiences of inequalities, people are often motivated to endorse attitudes that legitimize, justify and maintain that inequality [13–15]. The roles and the associated gender inequalities of childrearing might therefore foster endorsement of benevolent sexism. In contrast, an evolutionarily-informed account proposes that endorsement of benevolent sexism generates the conditions for reproductive success in heterosexual relationships, such as by promoting traditional mate preferences and adoption of traditional gender roles [7, 16, 17]. In sum, existing theories suggest a reciprocal relationship between individuals’ number of children and their endorsement of benevolent sexism that has been untested, likely due to the inherent methodological difficulties. The current research is the first to explore these associations in a preliminary way. We utilize a large national panel sample of New Zealanders (*N* = 6,017) to model the bidirectional lagged-effects of people’s number of children and their endorsement of benevolent sexism across a two-year span.

### Traditional gender beliefs, number of children, and postnatal inequalities

A key factor that links individuals’ reproductive outcomes with traditional gender beliefs is the increased gender inequalities that new parents experience. Women’s parental investment is significantly greater than that of men [i.e., gestation and lactation, 18], and parenthood magnifies gender inequalities in division of labor [e.g., 4, 5], career trajectories [2], and financial prospects [e.g., 19]. For example, mothers are more likely than fathers to make family accommodating changes at the expense of their career [20]. Across 36 countries comprising the Organization for Economic Co-operation and Development [OECD, 21], paternity leave is generally much shorter than maternity leave (on average 9 weeks vs 18.5 weeks) and sharable parental leave is predominantly used by mothers rather than fathers [1]. Across the world, new mothers experience sharp declines in working hours and income [19]; and the accompanying earning disadvantage appears to continue throughout the lifespan even in relatively egalitarian countries like New Zealand [3]. Thus, heterosexual parents are typically exposed to large, new gender inequalities.

Ambivalent sexism theory states that two ideologies—*hostile sexism* and *benevolent sexism*—function to legitimize and maintain gender inequalities [10, 16]. Hostile sexism consists of overtly negative attitudes toward women who challenge men’s power, such as career women [e.g., “Women seek to gain power by getting control over men”, 10]. Benevolent sexism consists of subjectively positive but patronizing attitudes toward women who support men’s power, such as women who prioritize their male partner’s career aspirations over hers (e.g., “A good woman should be set on a pedestal by her man”). Although the two types of sexism are consitently correlated, they differ in the ways of maintaining ineqaulities. Hostile sexism punishes women who challenge traditional gender roles, and benevolent sexism praises women who adopt traditional female roles and facilitate intimacy in romantic relationships [10, 16, 22]. As overtly sexist attitudes undermine men’s access to heterosexual intimacy, benevolent sexism is necessary to mask hostile sexism [10]. Thus, hostile and benevolent sexism together function as a reward-punishment system, with benevolent sexism being more appealing and prevelant than overt hostility [23].

These traditional gender beliefs idealize men and women as a cooperative unit with divided work duties in which men have a providing role and women are responsible for household maintenance and childcare [e.g., 6, 24–26]. The cooperative nature of heterosexual relationships—particularly those who have children—derives from the mutual dependency of sexual reproduction [18, 27]. Although gender roles are constrained by sex differences in reproduction, they are responsive to local conditions and supported by cultural beliefs [e.g., 6, 25, 28–30]. For example, the importance of cultural beliefs was highlighted in prior research showing that even under the same institutional arrangements in Switzerland, large cultural differences in beliefs about mothering exist between French vs. Germen speaking cantons [difference was 28.9 percentage point, 31, p.15]. Thus, idealizing gender relations as cooperative is a product of the coevolution of human mating strategies and our cultural heritage.

The reciprocal relationship between traditional gender roles—underpinned by cooperative gender beliefs—and reproductive outcomes is an assumed implication of prior research in psychology [10, 26]; sociology [17]; political science [32]; and economics [33]. Empirical studies have established a triangular pattern of associations between traditional gender attitudes and traditional partner preference with the adoption of traditional gender roles [i.e., provider vs. caregiver, 4, 8, 28, 34]; parenthood with traditional gender role-attitudes [35]; and parenthood with adoption of more traditional gender roles [4, 5, 36, 37]. These empirical studies are all consistent with the idea that people’s traditional gender beliefs are reciprocally linked with their adoption of traditional gender roles and their tendency of having more (vs. less) children. As we discuss next, the *direction* of this association is relevant to two complementary accounts on the sources and functions of benevolent sexism.

### Benevolent sexism as role justification: More children predicts benevolent sexism?

Two social psychological theories provide foundation for the expectation that the more children people have, the more they tend to endorse benevolently sexist beliefs. First, social role theory posits that social roles shape stereotypical gender beliefs [30]. Observing and adhering to social roles foster the development of beliefs about the typical characteristics of social groups [12], indicating that these beliefs are dynamic to the extent of which they reflect changes in social roles [11]. As parenthood is associated with changes in social roles [e.g., 4, 5, 36, 37], having children should promote the endorsement of gender attitudes that are relevant to people’s perceived suitability as parents [35]. Benevolent sexism, unlike hostile sexism, offers appealing stereotype contents for parents because it highlights women’s nurturing and caring abilities [10, 22].

A mutually compatible explanation suggests that the experience of the inherent gender inequalities in parenthood should prompt people to endorse sexist beliefs that justify those inequalities. System justification theory states that people have a strong motivation to preserve positive attitudes towards inequalities that appear to be persistent and inevitable. That is, both advantaged and disadvantaged groups are motivated to justify inequality to reduce the unpleasant feelings of unfairness, meaning that they can perceive social relations as fair, just and even desirable [14, 15, 38]. Indeed, people’s tendency to rationalize injustice increases as anticipated inequalities become their current reality [39], and they are more likely to rationalize conditions from which they cannot leave [40]. Benevolent sexism is a particularly appealing justification for gender inequality because it positively evaluates women in traditional gender roles as being “pure” and “morally superior” [10, 22, 24, 41]. Thus, both social role theory and system justification theory suggest that men and women with a greater number of children will endorse benevolent sexism more strongly.

### Benevolent sexism as a mating strategy: Benevolent sexism predicts more children?

A complementary perspective on the association between the number of children an individual has and their endorsement of benevolent sexism is derived from an evolutionarily-informed account of benevolent sexism. In a sex-role and gender-role divided society anticipated postnatal inequalities contribute to the development of cooperative gender beliefs that foster the conditions of maximizing men’s and women’s reproductive benefits [7, 25, 26]. Parenthood creates new inequalities undermining women’s societal status and increasing their interpersonal dependency [4, 19, 42, 43]. These anticipated inequalities indicate that *for women* securing a reliable male partner with traditional providing potentials; and respectively *for men*, signaling desirable male characteristics are still effective mating strategies [7, 25, 26]. Accordingly, across cultures, women place a higher importance on romantic partners’ dependability and stability compared to men [44, 45], and have prevailing preferences for partners’ providing capacity [46]. Endorsement of benevolent sexism signals traditional mate qualities reflecting traditional relationship roles [7, 8, 34], and offers women security, protection and commitment for fulfilling traditional role expectations [47, 48]. Indeed, even highly feminist women—being aware of the undermining effects of benevolent sexism (e.g., restricting their agency)—express relative preferences for men who endorse benevolent sexism as romantic partners compared to hostile sexism, ambivalent sexism or no sexism [49], because of their *perceived willingness to provide*—after accounting for other effects such as perceived warmth [7]. Thus, the mating strategy hypothesis suggests that both men and women who endorse benevolently sexist beliefs will show a greater tendency to have more children over time.

### Reciprocal links

These two theoretical perspectives are not mutually exclusive. In fact, they *both* emphasize that the structure of traditional heterosexual relationships, particularly those that have children, have inherent inequalities. These perspectives are complementary in a way that together they suggest a process in which (a) inequalities prompt justifying beliefs which then perpetuate those inequalities; and (b) encourage mating strategies which are based upon, and further lead to inequalities. On the other hand, people with fewer children may experience less inequalities, and consequently show lower tendencies to justify their circumstances. Accordingly, in the current research we conduct preliminary tests of whether people who have more children also tend to endorse benevolently sexist beliefs to a greater degree, and the potential for this association to be bidirectional across time.

### Current research

To assess the bidirectional relationship between individuals’ number of children and their endorsement of benevolent sexism, we conducted a cross-lagged analysis across a two-year period on a large panel sample of the relatively egalitarian country of New Zealand [N = 6,017; see 50]. Our rationale for the timespan was based on fertility research indicating that 92% and 82% of women in the age groups of 19–26 and 35-39-years-old, respectively, succeed to conceive within a year with regular intercourse at a frequency of twice per week [51]. More recent large-scale data also indicates that women in the top 10% of predicted probabilities have 88% chance of pregnancy over six menstrual cycles [52]. Existing research on the development of sexist attitudes also indicates that changes in endorsement of benevolent sexism are detectable over the timespan of 9-months to a year [53]. Thus, two years is a reasonable preliminary timespan to observe potential changes in the number of children people have and in their endorsement of benevolent sexism.

Our study makes two major contributions to the literature on benevolent sexism: First, we explore two theoretical accounts on the functions of benevolent sexism—which differ in their focus on one of the outcomes vs. one of the sources of gender inequalities—to build a comprehensive theoretical framework for the relationship between individuals’ reproductive outcomes and their endorsement of benevolent sexism. Second, we tentatively test this theoretical framework by assessing the time-lagged direction of the relationship between the number of children people have and their tendency to endorse benevolently sexist beliefs. Our quantitative study also extends prior experimental research on the link between stereotypical beliefs associated with social roles [e.g., 12], and the link between stereotype exposure and enhanced system justification [e.g., 13]. By utilizing a large panel sample, we provide a more naturalistic test whether having a greater number of children—as a proxy of stereotypical gender role performance within the family—is associated with endorsing higher levels of stereotypical beliefs two years later. Given the methodological difficulties of testing associations involving individuals’ number of children we tested our hypotheses in an exploratory fashion: For both men and women we expected a positive association between the number of children they have and their tendency to endorse benevolently sexist beliefs (Hypothesis 1). Following social role theory and system justification theory, we expected that having a greater number of children at Time 1 would predict a higher endorsement of benevolent sexism two years later, at Time 2 (Hypothesis 2). Following mating strategy theory, we expected that endorsing a higher level of benevolent sexism at Time 1 would predict having a greater number of children at Time 2 (Hypothesis 3).

## Materials and methods

### Data availability and ethics information

The data described in the paper are part of the New Zealand Attitudes and Values Study (NZAVS) [54]. Full copies of the NZAVS data files are held by all members of the NZAVS management team and advisory board. A de-identified dataset containing the variables analysed in this manuscript is available upon request from Chris Sibley (c.sibley@auckland.ac.nz), or any member of the NZAVS advisory board for the purposes of replication or checking of any published study using NZAVS data. The Mplus syntax used to test all models reported in this manuscript are available on the NZAVS website: www.nzavs.auckland.ac.nz. The NZAVS is reviewed every three years by the University of Auckland Human Participants Ethics Committee. The first phases of the longitudinal study were approved on 09-September-2009 until 09-September-2012, and renewed on 17-February-2012 until 09-September-2015 by The University of Auckland Human Participants Ethics Committee (Reference Number: 6171). Ethics approval for the study was re-approved on 03-June-2015 until 03-June-2018, and renewed on 05-September-2017 until 03-June-2021 (Reference Number: 014889). All participants granted informed written consent. Contact details are removed when the questionnaires are received, and all data were de-identified before analyses were conducted.

### Participants and procedure

The current study used data from Wave 4 (year = 2012; Time 1) and Wave 6 (year = 2014; Time 2). Individuals were posted a copy of the questionnaire from the New Zealand electoral-roll and sampled a total of 12,182 in Wave 4 and 15,822 in Wave 6 with a year-to-year retention rate of about 80%. We confined our analyses to 6,017 participants who provided full responses to the measures of our interests at both waves. Of the 6,017 participants, 3714 were women and 2303 men. Participants’ gender was measured as a binary variable because the ambivalent sexism inventory has exclusively been validated for heteronormative samples and there are no current validation studies demonstrating measurement invariance for LGBTQ+ samples [see 55]. 74.47% of women and 79.77% of men were parents in 2012, which grew up to 76.10% for women and 81.13% for men in 2014. Men on average had more children (*M* = 2.06, *SD* = 1.48) than women (*M* = 1.83, *SD* = 1.44) in 2012 and in 2014 (Men: *M* = 2.10, *SD* = 1.48; Women: *M* = 1.89, *SD* = 1.44). Parents’ level of benevolent sexism (*M* = 3.83, *SD* = 1.14) was higher than that of non-parents at Time 1 (*M* = 3.47, *SD* = 1.15) in 2012 [*F*(1, 1402.67) = 90.9292, *p* < .001, *ξ* = .22]; and at Time 2 (Parents: *M* = 3.84, *SD* = 1.16; Non-parents: *M* = 3.40, *SD* = 1.18; [*F*(1, 1263.73) = 127.753, *p* < .001, *ξ* = .27)]. Newly became parents at Time 2 (*n* = 92) also showed higher levels of benevolent sexism (*M* = 3.75, *SD* = 1.08) than those who stayed childless over the two years (*n* = 1322, *M* = 3.40, *SD* = 1.18), [*F*(1, 64.97) = 7.5993, *p* = .008, *ξ* = .21].

### Measures

#### Number of children

To assess people’s number of children, we used a single item “How many children have you given birth to, fathered, or adopted?”, ranging from 0–13. Our analyses were restricted to respondents aged 18–55 with an increase in number of children ranging between 0–3 over two years. We excluded 2 individuals reporting having 3.5 and 5.5. children; 3 individuals reporting having 6, 7, and 8 children between the two timepoints; 149 individuals reporting loss of children; and 51 individuals over the age of 55 reporting any increase in number of children. We imposed these restrictions because even if these reported numbers reflect reality, we did not expect our theoretical predictions to generalize to groups of people with unusual circumstances (e.g., losing a child). Of the 6,017 total cases, 289 people reported an increase in number of children (267 people reported to have one child; 20 people reported two; and 2 people reported to have three children), which yielded in a total of 313 additional children over two years.

#### Ambivalent sexism

Attitudes towards women were assessed using shortened five-item scales for each type of sexism from the Ambivalent Sexism Inventory [ASI, 10]; (1 = *Strongly Disagree* to 7 = *Strongly Agree*). Benevolent sexism (Cronbach’s α_T1_ = .73 and α_T2_ = .76) was measured using items 8, 9, 12, 19, and 22 of the ASI and hostile sexism (α_T1_ = .82 and α_T2_ = .82) was assessed using items 5, 11, 14, 15, and 16 of the ASI [for item wording, see 10].

#### Statistical covariates

As it is standard for research on ambivalent sexism, we statistically adjusted for people’s endorsement of hostile sexism when predicting benevolent sexism, and for benevolent sexism when predicting hostile sexism in an additional model [see 56]. We also included age, education, and household income as covariates given their established associations with gender attitudes and parenthood [e.g., 57, 58]. In a further additional analysis we assessed household income as a possible moderator of the bidirectional links between number of children and benevolent sexism because gender role attitudes among parents could be context dependent with financial resources constraining or enabling women to benefit from paid work and childcare arrangements [58].

## Results

To test the association between endorsement of benevolent sexism and number of children, first, we conducted a cross-sectional analysis at T1. Then, we conducted a structural equation model to examine the cross-lagged effects of endorsement of benevolent sexism and number of children across T1 and T2. Descriptive statistics and bivariate correlations are displayed in Table 1. As expected, benevolent sexism had a positive small correlation with number of children at both time points. Benevolent sexism at T1 had a strong positive correlation with benevolent sexism at T2. The correlation of number of children between T1 and T2 was extremely high due to the large majority of the sample not increasing in the number of children that they have. For the main analyses we first entered only the main predictors and gender interactions in the model, and in the second step we entered all the covariates with gender interactions. Accounting for gender variances is a standard practice in sexism research because there is an inherent measurement difference between women’s and men’s answers about gender beliefs due to their relative differences in societal roles and status. We therefore allowed each variable to vary by gender to control for any gender variances in the outcome variables. Statistical analyses were conducted in Mplus version 8.4 [59], with maximum likelihood estimation and bias-corrected confidence intervals estimated via 5,000 bootstrap draws.

**Table 1:** Descriptive statistics and bivariate correlations.

	1.	2.	3.	4.	5.	6.	7.	8.
1. Time 1 Age	-	-	-	-	-	-	-	-
2. Time 1 Education ^a^	-.18***	-	-	-	-	-	-	-
3. Time 1 Household Income ^b^	-.16***	.24***	-	-	-	-	-	-
4. Time 1 Hostile Sexism ^c^	.04***	-.22***	-.08***	-	-	-	-	-
5. Time 1 Benevolent Sexism ^c^	.12***	-.27***	-.11***	.42***	-	-	-	-
6. Time 1 Number of Children ^d^	.46***	-.16***	-.03*	.05***	.16***	-	-	-
7.Time 2 Benevolent Sexism ^c^	.14***	-.27***	-.12***	.38***	.74***	.17***	-	-
8. Time 2 Number of Children ^d^	.42***	-.15***	-.03*	.05***	.16***	.99***	.17***	-
Mean	50.79	5.04	102415.2	2.94	3.74	1.92	3.74	1.97
SD	14.20	2.84	94118.67	1.18	1.15	1.46	1.18	1.46

*Note*. *N* = 6,017; *df* = 6,538

*** *p* < .001

** *p* < .01

* *p* < .05

^a^ Education ranged from 0 (no qualification) to 10 (highest level of qualification)

^b^ Household Income was log-centred

^c^ Scale ranged from 1 (strongly disagree) to 7 (strongly agree)

^d^ Number of children ranged between 0–13; the correlations for number of children do not account for zero-inflation.

### Cross-sectional analysis

Results are displayed in Table 2. As expected, having a greater number of children was associated with endorsing a higher level of benevolent sexism. Results from the basic model indicated that this association was stronger for men than for women, however, after controlling for possible confounding effects, there was no evidence that this effect differed between men and women (*p =* .264). Hostile sexism was positively associated with benevolent sexism for both men and women, but more so for women than for men. There was no evidence that the main effect of age was related to benevolent sexism (*p* = .082), however it was moderated by gender in such that older men were slightly higher in benevolent sexism than older women (1 SD above the mean, slope *B* = 0.007, *SE* = 0.002, *p* < .001), but there was no gender difference in level of sexism among younger people [1 SD below the mean, *p* = .082; for detailed differences in sexist attitudes across age and gender, see 60]. Education and household income had a negative relationship with benevolent sexism, and there was no evidence that these effects were moderated by gender (*p* = .685 and *p* = .472 respectively). Thus, results from these analyses supported *Hypothesis 1*: there was a positive association between people’s endorsement of benevolent sexism and their number of children.

**Table 2:** Cross-sectional multiple regression models predicting benevolent sexism at T1.

	Benevolent Sexism–basic model				Benevolent Sexism–full model			
	B	SE	CI 2.5%	CI 97.5%	B	SE	CI 2.5%	CI 97.5%
Gender ^a^	0.097**	0.029	0.041	0.154	0.087**	0.029	0.031	0.144
Hostile Sexism ^b^ ^c^	0.464***	0.016	0.431	0.496	0.422***	0.017	0.389	0.456
Number of Children ^b^ ^d^	0.082***	0.012	0.057	0.106	0.070***	0.014	0.042	0.098
Hostile Sexism × Gender	-0.166***	0.026	-0.217	-0.115	-0.153***	0.027	-0.204	-0.101
Number of Children × Gender	0.054**	0.019	0.017	0.091	0.024	0.021	-0.018	0.066
Age ^b^					-0.003	0.001	-0.005	0.000
Education ^b^ ^e^					-0.064***	0.006	-0.076	-0.051
Household Income ^b^					-0.053*	0.023	-0.098	-0.01
Age × Gender					0.009***	0.002	0.005	0.014
Education × Gender					0.004	0.01	-0.015	0.023
H.Income × Gender					0.022	0.03	-0.041	0.081

*N* = 6,017

*** *p* < .001

** *p* < .01

* *p* < .05

^a^ Gender was contrast coded (0 = woman; 1 = man)

^b^ These variables were centred

^c^ Scale ranged from 1 (strongly disagree) to 7 (strongly agree)

^d^ Number of children ranged between 0–13

^e^ Education ranged from 0 (no qualification) to 10 (highest level of qualification); ^f^ Household income was log-centred.

### Cross-lagged analysis

In the second instance, we ran a structural equation model to predict the cross-lagged effects of endorsement of benevolent sexism and number of children across T1 and T2. In this model, benevolent sexism at T2 was one outcome and number of children at T2 was the other outcome. Results from these models are displayed in Table 3.

**Table 3:** Cross-lagged panel analysis predicting number of children and benevolent sexism over a two-year period.

	Number of Children Time 2				Benevolent Sexism Time 2			
	B	SE	CI 2.5%	CI 97.5%	B	SE	CI 2.5%	CI 97.5%
Gender ^a^	0.025	0.019	-0.008	0.066	0.101***	0.022	0.058	0.144
Age ^b^	0.004*	0.002	0.000	0.006	0.000	0.001	-0.002	0.002
Education ^b^ ^c^	-0.004	0.003	-0.010	0.000	-0.023***	0.005	-0.033	-0.012
Household Income ^b^ ^d^	0.073***	0.021	0.029	0.104	-0.041*	0.017	-0.077	-0.009
Benevolent Sexism T1 ^b^ ^e^	0.036	0.019	-0.003	0.061	0.687***	0.013	0.661	0.713
Hostile Sexism T1 ^b^ ^e^	-0.019	0.013	-0.038	0.010	0.086***	0.014	0.058	0.113
Number of Children T1 ^b^ ^f^	0.366***	0.028	0.331	0.428	0.033**	0.011	0.012	0.053
Age × Gender	0.000	0.002	-0.003	0.004	0.004*	0.002	0.001	0.007
Education × Gender	0.002	0.004	-0.006	0.011	-0.005	0.008	-0.020	0.010
H.Income × Gender	0.003	0.026	-0.042	0.058	0.028	0.024	-0.018	0.077
Benevolent Sexism × Gender	-0.029	0.021	-0.061	0.017	0.024	0.021	-0.016	0.064
Hostile Sexism × Gender	0.026	0.015	-0.009	0.049	-0.047*	0.021	-0.088	-0.006
Number of Children × Gender	-0.046	0.033	-0.114	0.009	-0.011	0.016	-0.042	0.019

*N* = 6,017

*** *p* < .001

** *p* < .01

* *p* < .05

^a^ Gender was contrast coded (0 = woman; 1 = man)

^b^ These variables were centred

^c^ Education ranged from 0 (no qualification) to 10 (highest level of qualification)

^d^ Household income was log-centred

^e^ Scale ranged from 1 (strongly disagree) to 7 (strongly agree)

^f^ Number of children ranged between 0–13.

#### Regression model predicting benevolent sexism

**Fig 1:**
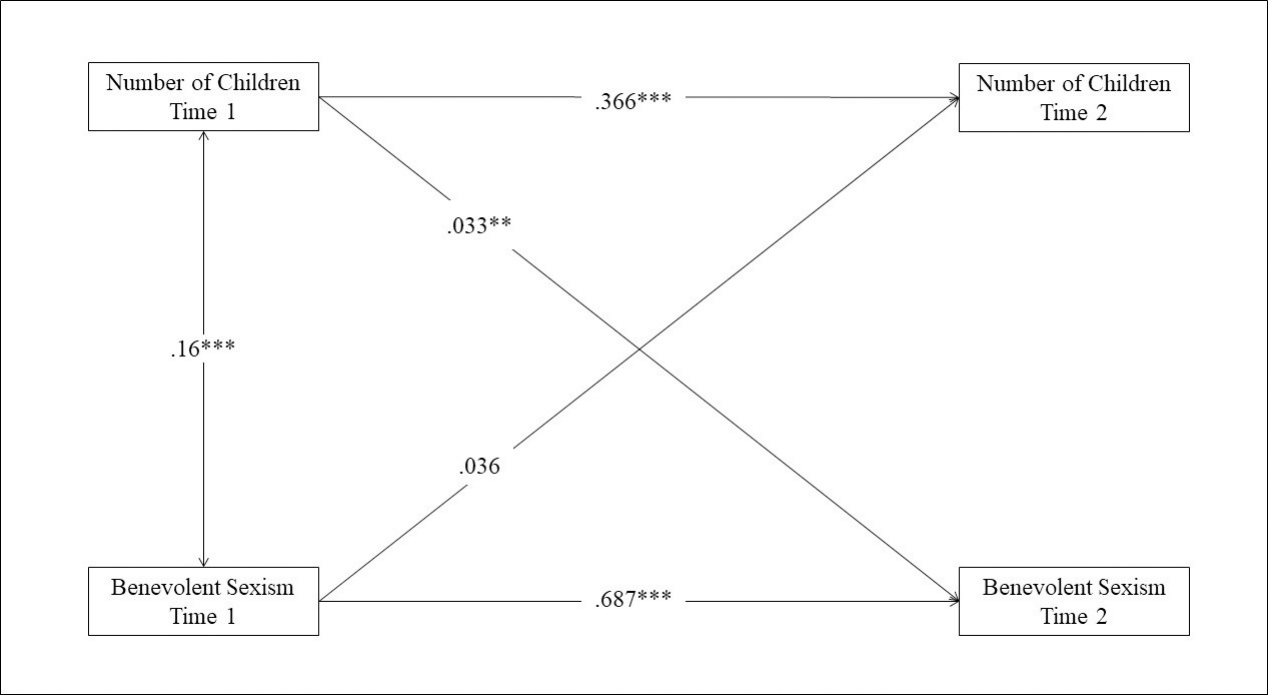
Cross-lagged panel analysis predicting number of children and benevolent sexism over a two-year time period while adjusting for the effects of covariates and the zero-inflation component of people’s number of children. (*N* = 6017, *** *p* < .001; ** *p* < .01).

In the first step we examined main effects without our covariates. Results indicated that having more children at T1 was associated with people’s greater endorsement of benevolent sexism at T2. In the next step we included our covariates which decreased the effect size of number of children on benevolent sexism, but the effect remained statistically significant; see Fig 1. Age was not a statistically significant predictor (*p* = .894). Education and household income were negatively, while hostile sexism was positively linked to benevolent sexism. Apart from age and hostile sexism, no evidence was found that any of the gender interactions were related to benevolent sexism (see Table 3). Thus, results from these analyses supported *Hypothesis 2*, indicating that having a greater number of children predicted a higher tendency to endorse benevolently sexist beliefs two years later.

#### Zero-inflated count model predicting residual variance in number of children

The excess of zeros in number of children at both time points was large (T1 *n* = 1414 and T2 *n* = 1322). Thus, the Vuong test [61] suggested a zero-inflated Poisson model to predict number of children. While adjusting for the zero-inflation in number of children—not presented—, results from the basic model indicated a weak positive relationship between benevolent sexism at T1 and number of children at T2 (*B* = 0.047, *SE* = 0.023, *p* = .039, *95% CI* [.001, .077]), yet this effect was no longer significant once we added our covariates into the model (*p* = .057). Only age and household income had a small positive relationship with number of children, and none of the gender interactions were statistically significant (see Table 3). Thus, these results did not provide support for *Hypothesis 3*, identifying no evidence in our sample that endorsement of benevolent sexism predicted people’s number of children over a two-year period when statistical covariates were considered.

### Additional analyses

We ran three additional models which are displayed in S1–S3 Tables. First, we ran our cross-lagged model without data restrictions in terms of age and number of children. This analysis included people in all ages and people who reported having more than three children over two years. Results remained essentially the same after lifting these restrictions; see S1 Table). Second, we added household income as a possible moderator of the bidirectional links between number of children and benevolent sexism. Results showed that household income did not moderate the relationship between benevolent sexism at T1 and number of children at T2 (*p* = .088), and its interaction with gender was not statistically significant (*p* = .066). Similarly, no statistically significant effects emerged for the opposite direction (*p* = .211), nor for the three-way interaction with gender (*p* = .200; see S2 Table). This finding indicated that effects of having children were no different across people with different levels of income. Finally, we assessed hostile sexism as an outcome variable in the cross-lagged model which included benevolent sexism as a covariate. Results showed no evidence that hostile sexism predicted number of children (*p* = .138), or that number of children predicted hostile sexism over two years (*p* = .331; see S3 Table). These results were particularly informative because it highlighted that the effects were specific to benevolent sexism, and not hostile sexism—which is consistent with the notion that the subjectively positive content of benevolent sexism makes benevolent sexism a more appealing and a more prevalent justification than overtly hostile sexist beliefs [e.g., 23].

## Discussion

We conducted the first analyses for the associations between individuals’ number of children and their endorsement of benevolent sexism across a two-year timespan. Supporting *Hypothesis 1*, a cross-sectional analysis indicated that people who had more children also tended to endorse benevolent sexism more strongly. We then conducted a structural equation analysis to explore the directionality of associations. Supporting *Hypothesis 2*, a greater number of children at one time point was associated with higher endorsement of benevolent sexism two years later. However, we did not identify any evidence that endorsement of benevolent sexism predicted a greater number of children two years later, thus not supporting *Hypothesis 3*. This study is the first to find preliminary evidence that there is an association between individuals’ number of children and their endorsement of benevolent sexism.

Our predictions were based on two theoretical accounts of the functions of benevolent sexism. First, a role justification account of benevolent sexism suggests that gender roles inform people’s beliefs about the skills and characteristics of women and men [12, 30]; and as individuals’ experiences of inequality increase with their investment in these roles, the more motivated they become to justify such inequalities [14, 15, 24]. Our findings supported this account. People’s greater number of children—an index of the extent to which people are experiencing gender inequalities [4, 35, 37]—was weakly related to their endorsement of benevolent sexism two years later. Also consistent with this theory, no gender differences emerged for this association.

That is, benevolent sexism is likely reflective of experiences of unequal gender roles associated with having a greater number of children, and thus, benevolent sexism could have a system-justifying function via idealizing the complementary nature of unequal divisions between men and women, while ensuring that their intimacy needs are met [10–12, 16, 17, 56, 62]. On the other hand, fewer children may reflect less inequalities and hence a lower need for justification. Thus, our research extends the literature on benevolent sexism theory, social role theory, and justification theory by modelling the lagged effects of having children on people’s benevolently sexist beliefs about women over two years in a large panel sample. Additionally, our results showed that effects were specific to benevolent sexism (vs. hostile sexism). This is consistent with ambivalent sexism theory suggesting that benevolent sexism explicitly praises women for their unequal prioritization of caregiver and childrearing roles, and flatters them into positively evaluating themselves as more suitable for subordinate roles, thereby reducing unpleasant feelings of unfairness [10, 14, 24].

Although justifying attitudes can palliate unpleasant feelings, rationalizing the status quo does not facilitate any positive change and hence it is not a constructive solution for inequalities [63, 64]. For example, the prevalent belief that mothers should be mainly concerned about childcare (vs. career), and that working women cannot establish a warm and secure relationship with their children have a strong negative effect on mothers’ tendency of re-entering the workforce and utilizing childcare support [65]. Thus, one of the real-world consequences of endorsing benevolently sexist beliefs could be increased inequalities stemming from strongly-gender typed parenting practices and women’s reduced labour force participation. That is, justification and related practices subsequently increase gender inequalities. Instead, to move forward, aiming for institutional solutions and nurturing cultural norms could foster a social environment in which having children does not widen gender gaps but allow equal work-sharing behavior for men and women [19, 66].

The second theoretical account we tested was that benevolent sexism functions as a mating strategy, encouraging men and women to adopt more traditional mate preferences and relationship roles (provider vs. caregiver), and thereby fostering conditions for reproductive outcomes [7, 26, 30, 34]. Importantly, any lack of an association does not provide evidence against this hypothesis. Our tentative tests for this association sought any potential evidence for this direction of effect. There may also be different facets relevant to reproductive outcomes that must be considered in research. First, traditional mate preferences and relationship roles may be related to the *quality* rather than *quantity* of reproductive outcomes. For example, stronger gender beliefs about the ‘provider vs. caregiver’ gender role divisions could serve children’s survival by increasing men’s greater financial investment; and women’s greater emotional investment in a few children, rather than having more children, which ultimately fosters the fitness of children. Second, we targeted a two-year timespan for the effects to manifest because previous fertility studies [51, 52], and research on sexism [53] indicated that fertility changes and development of sexist attitudes are detectable within two years. It is possible though, that the links between individuals’ sexist attitudes and their reproductive decisions unfold over longer periods of time.

Accordingly, the possibility of a bi-directional relationship between individuals’ sexist beliefs and people’s reproductive outcomes was not challenged by our findings that only supported one direction. We proposed that the role-justification and the mating strategy accounts of benevolent sexism are not rival perspectives but rather complementary explanations of a process in which inequalities in child-rearing practices prompt gender beliefs that justify inequalities, and these beliefs encourage mating strategies that recreate those inequalities. To further investigate the hypothesis that benevolent sexism facilitates conditions for successful reproduction, future research may investigate how benevolent sexism is related to the quality *and* quantity of reproductive outcomes over longer timespans.

### Caveats and future research directions

The purpose of this study was to tentatively test whether people who have more children also have a greater tendency to endorse benevolently sexist beliefs—a claim that has been long assumed but not investigated by previous research—making notable contributions to research on benevolent sexism. Theoretically, we mapped a comprehensive framework for how these associations may bidirectionally occur, highlighting that in the triangle of gender beliefs, gender inequality and parenthood there are reciprocal reinforcing relationships. Methodologically, we tested the associations between reproductive outcomes and benevolently sexist beliefs by modeling time lags to assess the directionality of effects. Thus, our study provided important preliminary insights into the association between individuals’ number of children and their endorsement of benevolent sexism, and points to possible directions for future research.

There were two method related limitations that future research should improve. First, although the core component of ambivalent sexism theory is heteronormativity, future sexism research should be extended to non-heteronormative people. It is currently not clear whether LGBTQ+ participants interpret sexism items in the same way as heteronormative participants, and whether the inventory measures the same thing in non-heteronormative contexts. For example, as discussed in Cowie, Greaves, and Sibley [55], it is possible, that although LGBTQ+ participants fall outside of the heteronormative gender framework and their interpretation of sexism measures differs from that of heteronormative people, LGBTQ+ still live in societies with a heteronormative gender system, and thus they might still be influenced by the same cultural factors as their heteronormative peers. Second, the broad representative sampling necessarily limited the number of scale items measuring benevolent sexism to five items. This precluded differentiating any potential effects of the different subfactors of benevolent sexism. For example, the subfactor of *heterosexual intimacy* (e.g., “Men are (not) complete without women”) should be particularly relevant for having children in heterosexual couples, but at the same time these measures are particularly problematic for measuring childrearing attitudes or tendencies in LGBTQ+ samples. Thus, future research should explore the particular relevance of heterosexual intimacy to understanding the link between sexism and having children by sampling from non-heteronormative populations and measuring benevolent sexism more fully.

Furthermore, due to the inherent methodological difficulties of conducting research on changes in individuals’ reproductive outcomes, data that can answer research questions on influential factors related to reproductive decisions over time are scarce. The residual-change model in our study does not directly account for the time-lapse between measurement points and is likely only generalizable to the 2-year time interval that we selected for our study [67]. Additionally, as people can realistically have one child over two years, the predicted residual change only suggests that benevolent sexism at Time 1 was associated with having one more child at Time 2 in our baseline model, indicating that results regarding the number of children people have should be interpreted with caution. In the future, large datasets with multiple measurement waves over a decade or more could also utilize growth-curve modeling to assess the trajectories of endorsement of benevolent sexism and reproductive outcomes over time. Thus, we encourage researchers to collect and utilize different forms of data to build up a collection of evidence for hypothesis-testing.

Establishing multi-method evidence is particularly essential because prior research suggests that the relationship between gender beliefs and parenthood is context-dependent, and should be shaped by individual-level experiences (e.g., availability of kin support in childcare) interacting with national-level structures that constrain individual choice [e.g., limited governmental childcare, 56, 64]. Future research should develop models that account for other individual-level factors, for example, having children could be indirectly linked to change in gender beliefs via women’s access to childcare.

Considering national-level factors, it is likely that people with more children have an even stronger tendency to endorse sexist beliefs in less (vs. more) egalitarian countries. System justification theory predicts that greater inequalities should prompt higher endorsement of justifying beliefs [13, 24], indicating that more extreme child-rearing inequalities (e.g., providing only maternity leave and not paternity leave) would lead to greater endorsement of benevolent sexism. Conversely, the mating strategy hypothesis suggests that the more gender segregated societies are, the more that people seek partners who fit into the ‘provider vs. caregiver’ model facilitating having more children [7, 26, 68]. Accordingly, benevolent sexism might be linked more strongly with successful reproductive outcomes in more traditional societies because mating preferences are more strongly tied to family-oriented roles [e.g., 6, 26]. Thus, future cross-cultural research should examine the association between individuals’ number of children and their endorsement of benevolent sexism as a function of national-level gender inequalities by using multilevel analyses in which country-level inequality measures are included as possible moderators.

## Conclusion

The present study examined the relationship between individuals’ number of children and their endorsement of benevolent sexism. We hypothesized a bidirectional positive association based on two accounts of how gender inequalities connect with gender ideologies. We found that having a greater number of children was weakly related to endorsing benevolently sexist beliefs more strongly two years later, but no evidence emerged linking individuals’ endorsement of sexist beliefs to the number of children one had over this timespan. Our study provides novel evidence that people’s number of children is linked with their endorsement of benevolent sexism, and new but small evidence for a direction of this relationship. These results contribute to existing research on benevolent sexism by testing the assumption that traditional gender role promoting beliefs are associated with reproductive outcomes. By developing a comprehensive theoretical framework and testing previously assumed links between number of children and benevolent sexism, our study is preliminary instructive for future research investigating these relationships.

## Supporting information

S1 Table Cross-lagged panel analysis predicting number of children and benevolent sexism over a two-year period without data restrictions. (DOCX)

S2 Table Cross-lagged panel analysis predicting number of children and benevolent sexism over a two-year period with household income as a moderator. (DOCX)

S3 Table Cross-lagged panel analysis predicting number of children and hostile sexism over a two-year period. (DOCX)
