# Supplementary material for: Individuals’ number of children is associated with benevolent sexism
Source: PLoS One. 2021 May 27;16(5):e0252194. doi: 10.1371/journal.pone.0252194 (PMC8158974; doi:10.1371/journal.pone.0252194)
Supplement: S3 Table — (DOCX) [file pone.0252194.s003.docx]

**S3 Table.** **Cross-lagged Panel Analysis Predicting Number of Children and Hostile Sexism over a two-year Period.**

|  | **Number of Children Time 2** | | | | **Hostile Sexism Time 2** | | | |
| --- | --- | --- | --- | --- | --- | --- | --- | --- |
|  | **B** | **SE** | **CI 2.5%** | **CI 97.5%** | **B** | **SE** | **CI 2.5%** | **CI 97.5%** |
| Gender ^a^ | 0.025 | 0.019 | -0.008 | 0.066 | 0.093*** | 0.022 | 0.051 | 0.136 |
| Age ^b^ | 0.004* | 0.002 | 0.000 | 0.006 | 0.002* | 0.001 | 0.000 | 0.004 |
| Education ^b c^ | -0.004 | 0.003 | -0.010 | 0.000 | -0.017*** | 0.005 | -0.027 | -0.008 |
| Household Income ^b d^ | 0.073*** | 0.021 | 0.029 | 0.104 | -0.008 | 0.013 | -0.034 | 0.017 |
| Benevolent Sexism T1 ^b e^ | 0.036 | 0.019 | -0.003 | 0.061 | 0.060*** | 0.013 | 0.035 | 0.086 |
| Hostile Sexism T1 ^b^ ^e^ | -0.019 | 0.013 | -0.038 | 0.010 | 0.704*** | 0.014 | 0.677 | 0.730 |
| Number of Children T1 ^b^ ^f^ | 0.366*** | 0.028 | 0.331 | 0.428 | 0.010 | 0.010 | -0.010 | 0.030 |
| Age × Gender | 0.000 | 0.002 | -0.003 | 0.004 | 0.000 | 0.002 | -0.004 | 0.003 |
| Education × Gender | 0.002 | 0.004 | -0.006 | 0.011 | -0.007 | 0.008 | -0.022 | 0.009 |
| H.Income × Gender | 0.003 | 0.026 | -0.042 | 0.058 | -0.012 | 0.023 | -0.059 | 0.032 |
| Benevolent Sexism × Gender | -0.029 | 0.021 | -0.061 | 0.017 | -0.044 | 0.022 | -0.088 | 0.000 |
| Hostile Sexism × Gender | 0.026 | 0.015 | -0.009 | 0.049 | 0.013 | 0.020 | -0.026 | 0.054 |
| Number of Children × Gender | -0.046 | 0.033 | -0.114 | 0.009 | -0.020 | 0.016 | -0.052 | 0.010 |

*N* = 6,017, *** *p*<.001; ** *p*<.01; * *p*<.05; ^a^ Gender was contrast coded (0 = woman; 1 = man); ^b^ These variables were centred; ^c^ Education ranged from 0 (no qualification) to 10 (highest level of qualification); ^d^ Household income was log-centred; ^e^ Scale ranged from 1 (strongly disagree) to 7 (strongly agree); ^f^ Number of children ranged between 0-13.
